# Odontiana

**Published:** 1888-11

**Authors:** 


ONDONTIANA.—It is during the eruption of the temporary teeth one observes troubles of a diverse nature, such as salivation, redness, (blushing), heat, and swelling of the gums, aphthæ, ulcerations, slight cough, diarrhœa, convulsions, fever with agitation, etc. Then interfere and make, if there is reason, an opening for the eruption of the teeth. When there is fever, give a solution of bi-carbonate of potash combined with a little citric acid, to which add some drops of tincture of henbane, in case of agitation.—DR. MACARIO in *Nice Medical.*

